# 1,4‐Dioxane‐degrading consortia can be enriched from uncontaminated soils: prevalence of *Mycobacterium* and soluble di‐iron monooxygenase genes

**DOI:** 10.1111/1751-7915.12850

**Published:** 2017-10-06

**Authors:** Ya He, Jacques Mathieu, Marcio L.B. da Silva, Mengyan Li, Pedro J.J. Alvarez

**Affiliations:** ^1^ Department of Civil and Environmental Engineering Rice University Houston TX 77005 USA; ^2^ Department of Chemistry and Environmental Science New Jersey Institute of Technology Newark NJ 07102 USA

## Abstract

Two bacterial consortia were enriched from uncontaminated soil by virtue of their ability to grow on 1,4‐dioxane (dioxane) as a sole carbon and energy source. Their specific dioxane degradation rates at 30°C, pH = 7 (i.e. 5.7 to 7.1 g‐dioxane per g‐protein per day) were comparable to those of two dioxane‐metabolizing archetypes: *Pseudonocardia dioxanivorans* CB1190 and *Mycobacterium dioxanotrophicus* PH‐06. Based on 16S rRNA sequencing, *Mycobacterium* was the dominant genus. Acetylene inhibition tests suggest that dioxane degradation was mediated by monooxygenases. However, qPCR analyses targeting the tetrahydrofuran/dioxane monooxygenase gene (*thmA/dxmA*) (which is, to date, the only sequenced dioxane monooxygenase gene) were negative, indicating that other (as yet unknown) catabolic gene(s) were responsible. DNA sequence analyses also showed threefold to sevenfold enrichment of group 5 and group 6 soluble di‐iron monooxygenase (SDIMO) genes relative to the original soil samples. Whereas biodegradation of trace levels of dioxane is a common challenge at contaminated sites, both consortia degraded dioxane at low initial concentrations (300 μg l^−1^) below detectable levels (5 μg l^−1^) in bioaugmented microcosms prepared with impacted groundwater. Overall, this work shows that dioxane‐degrading bacteria (and the associated natural attenuation potential) exist even in some uncontaminated soils, and may be enriched to broaden bioaugmentation options for sites experiencing insufficient dioxane catabolic capacity.

## Introduction

1,4‐Dioxane (dioxane) is a cyclic ether that is widely used as a stabilizer for chlorinated solvents, especially for 1,1,1‐trichloroethane (1,1,1‐TCA). Dioxane can be present as a solvent in paints, as well as in cosmetics and detergents (Zenker *et al*., 2003; Mohr *et al*., 2010), and it was classified as a probable human carcinogen (Class B2) by the United States Environmental Protection Agency (U.S. EPA) (U.S.EPA, 2014). As a result, some states have limited dioxane concentrations in drinking water to ≤ 0.35 μg l^−1^ based on 1 × 10^−6^ cancer risk assessments (U.S.EPA, 2014). Removal of dioxane from contaminated sites is usually challenging because of the compound's physical and chemical characteristics, including extreme water miscibility, limited volatility and low tendency to be retarded by sorption (Zenker *et al*., 2003).

Different remediation strategies have been proposed to remediate dioxane‐impacted sites, including advanced oxidation processes (Ikehata *et al*., 2016), photocatalytic degradation (Barndõk *et al*., 2016), phytoremediation (Aitchison *et al*., 2000), bioaugmentation (Isaka *et al*., 2016) and biostimulation (Lippincott *et al*., 2015; Li *et al*., 2017). Among these approaches, advanced chemical oxidation may offer effective source zone treatment, but with relatively high energy consumption and operational costs. Conversely, biodegradation approaches (when applicable) are more economical and environmentally friendly, and can also lead to dioxane mineralization without the accumulation of toxic by‐products.

Although dioxane is relatively recalcitrant, a number of strains capable of degrading dioxane have been isolated (Table 1). Most of these strains degrade dioxane cometabolically [i.e. dioxane degradation is induced by another substrate, and it does not serve as carbon or energy source to support bacterial growth (Alvarez and Illman, 2006)]. Nearly all of these strains were isolated from wastewater treatment plants or dioxane‐impacted sites. However, the potential for encountering dioxane degraders in uncontaminated environments, which is important to assess the distribution and potential ubiquity of dioxane degraders and the associated natural attenuation potential, has not been reported in the literature.

**Table 1:** Bacteria capable of metabolizing or cometabolizing dioxane

Bacterial strain	Dioxane metabolism^a^	Cosubstrate	Inoculum source	References
*Rhodococcus ruber* 219	+	−	WWTP effluent	(Bernhardt and Diekmann, 1991)
*Pseudonocardia dioxanivorans* CB1190	+	−	Industrial sludge from a dioxane contaminated site	(Parales *et al*., 1994)
*Pseudonocardia benzenivorans* B5	+	−	Soil contaminated by various chlorinated, aromatic compounds	(Kämpfer and Kroppenstedt, 2004)
*Mycobacterium sp*. PH‐06	+	−	River sediment contaminated by dioxane	(Kim *et al*., 2009)
*Afipia* sp. D1	+	−	Soil samples near a dioxane producing factory	(Sei *et al*., 2013b)
*Mycobacterium* sp. D6	+	−	Same as above	(Sei *et al*., 2013b)
*Mycobacterium* sp. D11	+	−	Same as above	(Sei *et al*., 2013b)
*Pseudonocardia* sp. D17	+	−	Same as above	(Sei *et al*., 2013b)
*Acinetobacter baumannii* DD1	+	−	WWTP sludge	(Huang *et al*., 2014)
*Rhodanbacter* AYS5	+	−	Industrial sludge	(Pugazhendi *et al*., 2015)
*Xanthobacter flavus* DT8	+	−	Activated sludge of pharmaceutical plants	(Chen *et al*., 2016)
*Pseudonocardia tetrahydrofuranoxydans* sp. K1	−	Tetrahydrofuran	WWTP sludge	(Kohlweyer *et al*., 2000)
*Pseudonocardia* sp. ENV478	−	Tetrahydrofuran	Industrial wastewater treatment system	(Vainberg *et al*., 2006)
*Rhodococcus ruber* T1	−	Tetrahydrofuran	Landfill soil	(Sei *et al*., 2013a)
*Rhodococcus ruber* T5	−	Tetrahydrofuran	WWTP Sludge	(Sei *et al*., 2013a)
*Flavobacterium* sp.	−	Tetrahydrofuran	A contaminated groundwater plume	(Sun *et al*., 2011)
*Mycobacterium* sp. JOB5	−	Propane	Soil samples with hydrocarbons	(Ooyama and Foster, 1965)
*Mycobacterium sp*. ENV421	−	Propane	Turf soil enriched with propane	(Masuda, 2009)
*Rhodococcus ruber* ENV 425	−	Propane	Turf soil samples enriched with propane	(Steffan *et al*., 1997)
*Pseudomonas mendocina* KR1	−	Toluene	Algal‐bacterial mat from Colorado River in Austin	(Whited and Gibson, 1991)
*Rhodococcus* RR1	−	Toluene	Gasoline contaminated aquifer	(Stringfellow and Alvarez‐Cohen, 1999)
*Ralstonia pickettii* PKO1	−	Toluene	Soil microcosms amended with BTEX	(Kukor and Olsen, 1990)
*Burkholderia cepacia* G4	−	Toluene	Water and soil samples	(Nelson *et al*., 1986)
*Methylosinus trichosporium* OB3b	−	Methane	Mud, river and stream water, soil samples	(Whittenbury *et al*., 1970)

WWTP, wastewater treatment plant; BTEX, benzene, toluene, ethylbenzene and xylenes.

**a.** ’+’ and ‘−’ indicate this condition is applicable and not applicable respectively.

The critical role of monooxygenases in dioxane degradation has been emphasized by several studies (Mahendra and Alvarez‐Cohen, 2006; Kim *et al*., 2009), and one gene cluster that is responsible for dioxane degradation by *Pseudonocardia dioxanivorans* CB1190 has been identified and sequenced (i.e. *thmADBC*) (Sales *et al*., 2011). This gene cluster codes for dioxane monooxygenase, which is a soluble di‐iron monooxygenase (SDIMO) that initiates dioxane degradation (Grostern *et al*., 2012). SDIMOs are multicomponent bacterial enzymes that also catalyse the oxidation of various priority pollutants such as chlorinated solvents, aromatic hydrocarbons, alkanes and alkenes (Notomista *et al*., 2003). Therefore, investigating the presence and diversity of SDIMO genes in various environments is important to advance understanding of intrinsic bioremediation potential.

In this study, we describe the enrichment of two consortia from garden soils with no known previous exposure to dioxane. We compare their dioxane degradation capabilities with those of two archetype degraders [*Pseudonocardia dioxanivorans* CB1190 (Parales *et al*., 1994) and *Mycobacterium dioxanotrophicus* PH‐06 (Kim *et al*., 2009; He *et al*., 2017)]. Both high (500 mg l^−1^) and low (300 μg l^−1^) initial dioxane concentrations are considered to assess the performance of these consortia over a broad range of scenarios at impacted sites. We also examine the microbial communities before and after enrichment with dioxane through phylogenetic gene sequencing analyses, and investigate the suspected enzymes that initiate the degradation process using acetylene inactivation tests (Prior and Dalton, 1985), qPCR assays and SDIMO sequence analyses (Coleman *et al*., 2006).

## Results and discussion

### Dioxane degradation rates for both consortia were similar to those of two archetype degraders

**Figure 1:**
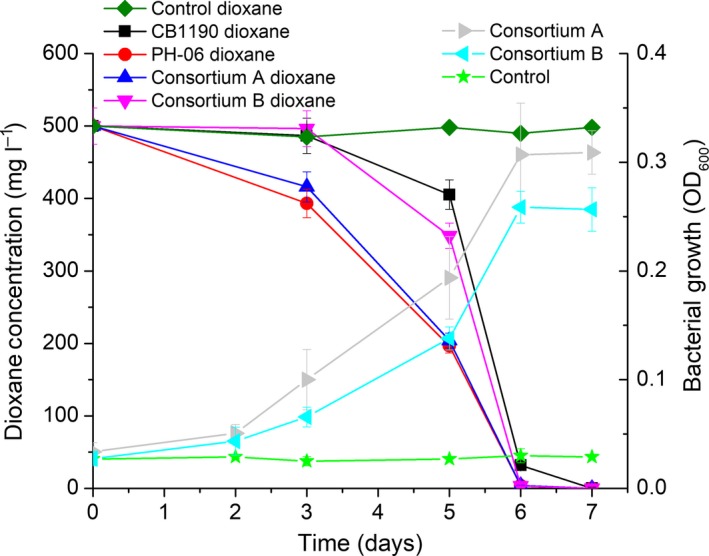
Dioxane degradation by enriched consortia and two archetypes (left) and concurrent growth of consortia (right). Experiments were conducted using AMS medium with an initial dioxane concentration of 500 mg l^−1^. The initial biomass densities (measured as protein content) were 25 mg l^−1^ for CB1190, 32 mg l^−1^ for PH‐06, 19 mg l^−1^ for Consortium A and 36 mg l^−1^ for Consortium B. The controls contained autoclaved bacteria.

Using surface soil samples (5 g) with no known history of dioxane exposure as bacterial seeds, two consortia were enriched over 3 months following 12 weekly cycles of 10‐fold serial dilution and growth in 100 ml of ammonium mineral salts (AMS) medium (Parales *et al*., 1994; Li *et al*., 2010) amended with 100 mg l^−1^ dioxane as sole carbon source. These two consortia were capable of degrading 500 mg l^−1^ dioxane (in AMS medium) as sole source of carbon and energy to non‐detect levels (< 5 μg l^−1^) within 1 week (Fig. 1), with a doubling time of approximately 24 h at 30°C and pH = 7. After normalizing rates to the biomass concentrations, the specific degradation rates were 7.12 ± 0.18 g‐dioxane per g‐protein per day for Consortium A and 5.68 ± 0.07 g‐dioxane per g‐protein per day for Consortium B. These rates are comparable to those of two archetype dioxane degraders, *Pseudonocardia dioxanivorans* CB1190 (6.21 ± 0.68 g‐dioxane per g‐protein per day) and *Mycobacterium dioxanotrophicus* PH‐06 (7.35 ± 0.20 g‐dioxane per g‐protein per day) (Fig. 1). For both consortia, dioxane removal was coupled to bacterial growth (cell yield coefficients were 0.11 g‐protein per g‐dioxane for Consortium A and 0.09 g‐protein per g‐dioxane for Consortium B) (Fig. 1), indicating metabolic (rather than cometabolic) degradation of dioxane as sole carbon and energy source.

**Figure 2:**
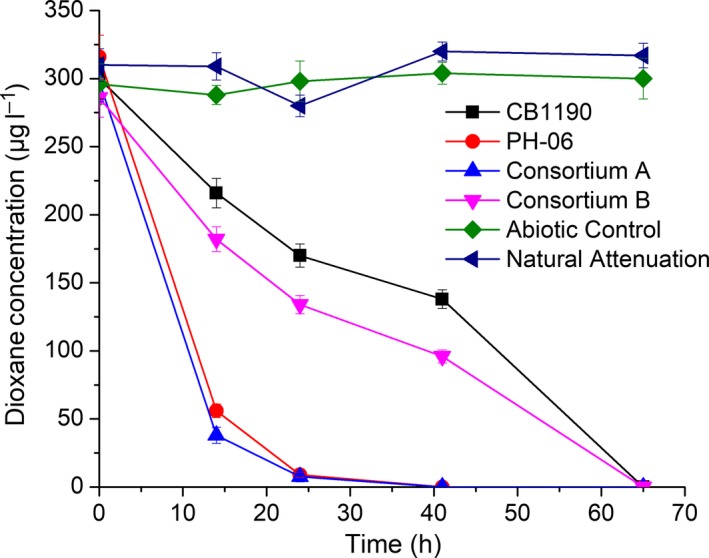
Dioxane degradation in various bioaugmented microcosms. Experiments were conducted using groundwater (initial dioxane concentration was 300 µg l^−1^) from a dioxane‐impacted site in Seattle, Washington, USA, T = 15°C. The added bacterial biomass measured as protein content was 20 mg l^−1^ for CB1190, 32 mg l^−1^ for PH‐06, 48 mg l^−1^ for Consortium A and 18 mg l^−1^ for Consortium B.

Dioxane‐impacted groundwater from a site in Seattle, WA, that was not exhibiting intrinsic biodegradation capabilities was used to assess the potential of these consortia for bioaugmentation. Addition of both consortia (48 mg l^−1^ for Consortium A and 18 mg l^−1^ for Consortium B) resulted in dioxane removal below detection levels (< 5 μg l^−1^) within 3 days, even when the initial dioxane concentration was low (i.e. 300 μg l^−1^) (Fig. 2). This is an encouraging result because such low dioxane concentrations may represent a bioremediation challenge at impacted sites, as they might be insufficient to induce and/or sustain an active degrading indigenous population (Adamson *et al*., 2014; Rodriguez, 2016). The specific dioxane biodegradation rates of these two consortia (0.062 ± 0.003 g‐dioxane per g‐protein per day for Consortium A and 0.056 ± 0.003 g‐dioxane per g‐protein per day for Consortium B) are comparable with those of two archetype degraders (0.052 ± 0.002 g‐dioxane per g‐protein per day for CB1190 and 0.10 ± 0.005 g‐dioxane per g‐protein per day for PH‐06) when the initial dioxane concentration was similarly low.

A key challenge to consider in bioaugmentation is the subsurface distribution of injected bacteria, which is hindered by filtration through the aquifer material – especially when the cultures aggregate. This is an important limitation for bioaugmentation with the archetype dioxane degraders CB1190 and PH‐06, which form clumps in suspension (Kim *et al*., 2009; Grostern *et al*., 2012). Interestingly, these two consortia do not clump as much as CB1190 and PH‐06 (Fig. S1), which could make them superior candidates for *in situ* bioaugmentation (i.e. easier perfusion through porous media) at sites where the indigenous dioxane biodegradation capacity is insufficient. Furthermore, this unprecedented enrichment of two consortia from uncontaminated soil based on their capability to grow on dioxane as sole carbon source indicates that pristine environments may harbour dioxane degraders and exhibit associated natural attenuation capacity.

Dioxane is a synthetic organic (rather than naturally occurring) compound to which the original soil biota had no known previous exposure. Thus, the observed metabolic capacity may seem counterintuitive. Nevertheless, all reported dioxane‐degrading bacteria can also degrade other cyclic ethers such as tetrahydrofuran (THF) and some naturally occurring compounds. For example, cyclic ethers containing 5‐ and 6‐membered rings are widespread in nature and are used in the biosynthesis of certain antibiotics (Rutkowski and Brzezinski, 2013; Martín *et al*., 2014), and the capacity to degrade these antibiotics may serve a protective role (Topp *et al*., 2013). Additionally, lactones, which can be intermediates of both dioxane and THF biodegradation (Bernhardt and Diekmann, 1991; Sales *et al*., 2013) (Fig. S2), are also commonly encountered in nature as chemical signals for bacterial communication (i.e. quorum sensing) (Keller and Surette, 2006). Many bacteria produce lactonases to degrade these signals and interrupt quorum sensing (Fig. S2), which enhances their ability to compete against the bacteria that produce them (Dong *et al*., 2001; Wang *et al*., 2004). This raises the possibility that biodegradation of simple cyclic ethers such as THF and dioxane (which are lactone precursors) may be a fortuitous result of backward evolution of catabolic pathways (Caetano‐Anollés *et al*., 2009). Specifically, we cannot rule out the possibility that if an organism is capable of growing on lactones that became depleted, this could impose selective pressure to transform other compounds (e.g. dioxane) into a lactone for enhanced survival.

### Microbial characterization in both dioxane‐degrading consortia

**Figure 3:**
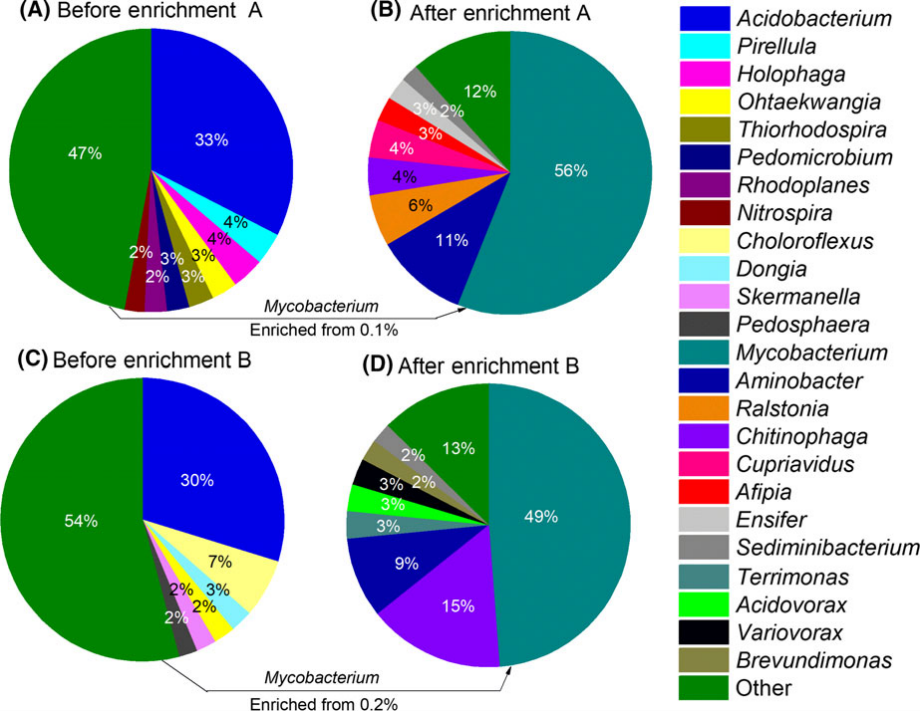
16S rRNA sequencing analyses of the two original soil samples and enriched consortia. The sum of all bacteria genera representing < 2% of total community is indicated as ‘Other’. These sequence data were submitted to the GenBank database under accession number SRP103870.

Phylogenetic analysis by 16S rRNA sequences revealed a decrease in microbial diversity after enrichment on dioxane. For Consortium A, the number of genera present decreased from 288 in the original soil samples to 47 genera after enrichment. The corresponding decrease in Consortium B was from 340 to 50. The dominant genus in the original soils samples was *Acidobacterium* (33% in Consortium A and 30% in Consortium B) (Table 2). After enrichment, the dominant genus in both consortia is *Mycobacterium* (56% in Consortium A and 49% in Consortium B) (Fig. 3). Enrichment significantly changed the microbial community structure. For example, the top three genera in the original soil samples disappeared, while the top 3 genera in enriched consortia were minor populations in the original soil samples (Table 2). The observed population shift with a clear predominance of *Mycobacterium* spp., suggests that some species from this genus were involved in dioxane biodegradation.

**Table 2:** Three most abundant genera before and after enrichment

Consortium A			Consortium B		
Genus name	Percentage (%)		Genus name	Percentage (%)	
	Before	After		Before	After
*Acidobacterium*	**31.9** a	0.00	*Acidobacterium*	**29.81**	0.00
*Pirellula*	**3.6**	0.00	*Chloroflexus*	**6.67**	0.00
*Holophaga*	**3.6**	0.00	*Dongia*	**2.56**	0.00
*Mycobacterium*	0.1	**56.0**	*Mycobacterium*	0.2	**48.8**
*Aminobacter*	0.04	**10.5**	*Chitinophaga*	0.01	**15.5**
*Ralstonia*	0.08	**5.9**	*Aminobacter*	0.03	**9.2**

a Figure in bold represents significant change before and after enrichment.

Various *Mycobacterium* strains capable of degrading dioxane metabolically or cometabolically have been previously isolated from environmental samples with known dioxane exposure history (Table 1). The predominance of *Mycobacterium* spp, in these consortia supports the notion that this is an important and commonly encountered genus of dioxane degraders. Furthermore, we show that *Mycobacterium* may be more prevalent in some environments than the more widely studied dioxane‐degrading actinomycetes belonging to *Pseudonocardia* and *Rhodococcus* genera.

Among other genera present that make at least 2% of these consortia (Fig. 3) (i.e. *Aminobacter* (10%), *Ralstonia* (6%), *Chitinophaga* (4%), *Cupriavidus* (4%), *Afipia* (3%), *Ensifer* (3%) and *Sediminibacterium* (2%) in Consortium A; *Chitinophaga* (16%), *Aminobacter* (9%), *Terrimonas* (3%), *Acidovorax* (3%), *Variovorax*(3%), *Brevundimonas* (2%) and *Sediminibacterium* (2%) in Consortium B), only *Afipia* encompasses a strain (i.e. *Afipia* sp. D1) that was reported to degrade dioxane as sole carbon and energy source (Sei *et al*., 2013b). *Aminobacter* and *Sediminibacterium* were the only genera that were enriched in both consortia, with *Aminobacter* reaching much higher abundance. It is still uncertain if and how the other genera identified in these consortia contribute to the biodegradation of dioxane and/or its potential by‐products. Our unsuccessful efforts to isolate dioxane‐degrading strains using the conventional enrichment and dilution to extinction approach (Xing *et al*., 2010) suggest that the dominant *Mycobacteria* and other coexisting bacterial species might constitute complex syntrophic communities.

### Acetylene inhibition test indicates the involvement of monooxygenases

Acetylene, a known monooxygenase inhibitor (Prior and Dalton, 1985), was used to determine whether monooxygenases could play a role in dioxane degradation by the two consortia. When exposed to 8% (v/v) of acetylene in headspace, dioxane removal significantly decreased (by > 90% over 7 days) compared to the untreated controls (Fig. S3). These results suggest that monooxygenases played a crucial role on dioxane degradation, likely catalysing the initial biotransformation steps. This corroborates previous reports about the importance of monooxygenases in initiating the aerobic degradation of dioxane (Mahendra and Alvarez‐Cohen, 2006; Li *et al*., 2013b). Therefore, information on the responsible monooxygenase genes can advance understanding and assessment/ of dioxane biodegradation and natural attenuation potential at contaminated sites.

### The thmA/dxmA biomarker was not detected in these two consortia

The acetylene inhibition test indicates the involvement of monooxygenases, although it is still unclear which specific monooxygenases initiated dioxane catabolism. Up to date, despite the successful isolation of numerous dioxane‐degrading bacteria in previous work (Table 1), little is known about the genetic basis for dioxane biodegradation. In fact, only one enzyme responsible for initiating dioxane metabolism has been discovered. This tetrahydrofuran monooxygenase/dioxane monooxygenase is encoded by the gene cluster *thmADBC*, which is located on plasmid pPSED02 in *Pseudonocardia dioxanivorans* CB1190 (Grostern *et al*., 2012; Sales *et al*., 2013). A primer/probe set for the *thmA/dxmA* biomarker designed to target the nucleotide sequence coding for the hydroxylase alpha subunit (i.e. the enzyme's active site) was shown to be an excellent indicator of dioxane degradation capacity (Li *et al*., 2013a), and no false positives have been observed. However, this *thmA/dxmA* biomarker was not detected in either of our two consortia using qPCR analysis (limit of detection = 12 copies per ng genomic DNA; 16S rRNA from the consortia and *thmA/dxmA* from CB1190 were used as positive controls, Fig. S4). Therefore, other monooxygenase genes were likely responsible for dioxane degradation in these consortia.

### The role of soluble di‐iron monooxygenase (SDIMO) on dioxane biodegradation

A nested PCR analysis using two degenerate primers to target the hydroxylase alpha subunit of SDIMOs (Coleman *et al*., 2006) was performed to gain insights into which enzymes were responsible for dioxane degradation by these consortia. SDIMOs were present in both consortia as indicated by agarose gel electrophoresis (Fig. S5).

**Figure 4:**
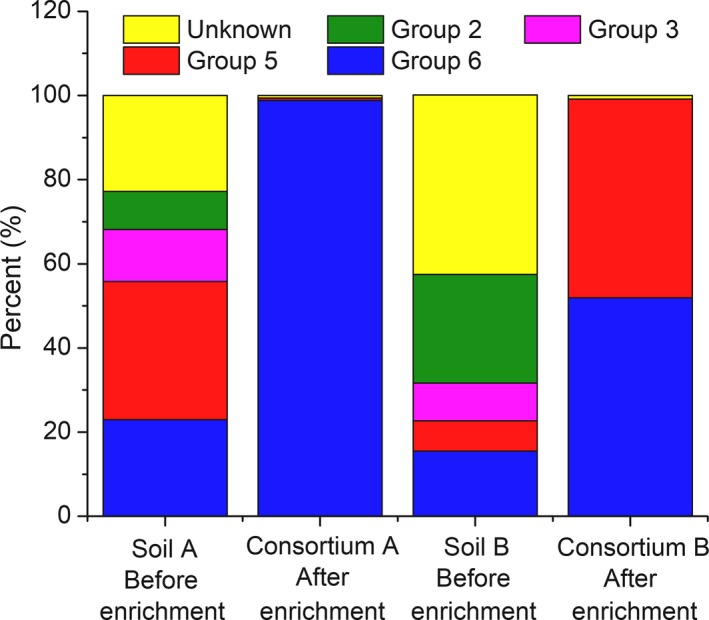
SDIMO gene sequencing of nested PCR products from the original soil samples and enriched consortia. ‘Group 2′, ‘Group 3′, ‘Group 5′ and ‘Group 6′ indicate a high similarity (> 93%) with groups 2, 3, 5 and 6 soluble di‐iron monooxygenase hydroxylase (SDIMOs) alpha subunit genes respectively. ‘Unknown’ indicates no significant similarities with any SDIMO gene sequences available on NCBI. The percentages bars represent the fractions of different SDIMO gene groups in the two consortia. These sequence data were submitted to the GenBank database under accession number SRP103872.

Soluble di‐iron monooxygenases are divided into six groups, based on their subunit organization and composition, substrate specificity and sequence similarity (Coleman *et al*., 2006), and the only sequenced dioxane monooxygenase gene cluster to date (i.e. *thmADBC*) belongs to group 5 (Grostern *et al*., 2012). To gain insight about the specific SDIMOs harboured by these two consortia, the purified products obtained from the second PCR run were sequenced and compared to genes databases (NCBI) encompassing all of the currently reported SDIMOs. The diversity of SDIMO genes in both consortia decreased compared with the original soil samples. The dominant SDIMO genes in Consortium A corresponded to a group 6 SDIMO (98.81%); while in Consortium B, SDIMO genes from both groups 5 (47.27%) and 6 (51.88%) were observed. These represent threefold to sevenfold increases, respectively, relative to the original soil samples (Fig. 4). In both consortia, the relative abundance of *thmA/dxmA* gene was negligible (0.03%), which is consistent with the negative amplification of these genes by qPCR (Fig. S4).

The prevalence of such SDIMOs is consistent with the observed enrichment of *Mycobacterium* spp., as previous studies have reported that groups 5 and 6 SDIMO genes are harboured by *Mycobacterium* spp. These include group 5 SDIMO genes in *Mycobacterium semegmatis* MC^2^ 155 and *Mycobacterium goodii* strain 12523 (Furuya *et al*., 2011), and group 6 SDIMO genes in *Mycobacterium chubuense* NBB4 (Coleman *et al*., 2006, 2011), *Mycobacterium* sp. TY‐6 (Kotani *et al*., 2006) and *Mycobacterium* sp. ENV421 (Masuda *et al*., 2012). This underscores the need for further research on SDIMO genes and enzymes involved in dioxane biodegradation to develop novel biomarkers that can be useful for forensic analysis and performance assessment of bioremediation and natural attenuation at dioxane‐impacted sites.

Overall, these data suggest that dioxane catabolic capacity may be found even in some uncontaminated soils and that such bacteria can be enriched from such soils to broaden bioaugmentation applications to bioremediate sites experiencing negligible dioxane degradation potential.

## Experimental procedures

### Enrichment of dioxane‐degrading bacteria consortia

Two dioxane‐degrading bacteria consortia (designated as Consortium A and Consortium B) were enriched from garden soil that had no known previous exposure to dioxane. Soil samples were obtained from the Rice University campus (Houston, TX, USA) at a depth of 5 cm from the soil surface. Two samples (corresponding to the two consortia) were collected at two different spots (10 m away) at different seasons (in September 2015 and March 2016). A dark soil sample collected from an area near bushes and trees was used for the enrichment of Consortium A, while a lighter yellowish soil sample from a grassy area was used to enrich Consortium B. No dioxane was detected in these soils (detection limit < 5 μg l^−1^).

Soil samples (5 g of soil in 10 ml of deionized water) were vortexed vigorously on a Vortex‐Genie^®^ 2 (MO BIO Laboratories, Inc. Carlsbad, CA, USA, at the maximum speed for 10 min, and the aqueous phase was used as the initial inoculum of the enrichment. A series of 10‐fold dilutions (Xing *et al*., 2010) were performed for each sample each week, using ammonium mineral salts (AMS) medium (Parales *et al*., 1994; Li *et al*., 2010) amended with 100 mg l^−1^ of dioxane as the sole source of carbon. Amphotericin B solution (2.5 mg l^−1^) (Sigma‐Aldrich, St Louis, MO, USA) was added to the medium to avoid the growth of fungus, which could confound assessment of dioxane biodegradation by bacteria. All samples were kept at 30°C while shaking on a Digital Linear Shaker (Scilogex, Rocky Hill, CT, USA) at 150 rpm.

After 12 cycles of enrichment for approximately 3 months, rapid dioxane biodegradation was sustained at relatively constant rates by both consortia. However, isolation of pure strains that metabolize dioxane was unsuccessful using agar plates prepared with 1.5% molecular grade agar and AMS amended with 100 mg l^−1^ of dioxane. The failure to isolate dioxane degraders was partially due to the fact that some bacteria in the enriched consortia grew on the plates using agar, rather than dioxane, as carbon source. Thus, the enriched consortia were used for further studies.

### Dioxane degradation rates and bioaugmentation studies

Dioxane degradation capabilities by these consortia were benchmarked against those of two archetype degraders: *Pseudonocardia dioxanivorans* CB1190 (Parales *et al*., 1994) and *Mycobacterium dioxanotrophicus* PH‐06 (Kim *et al*., 2009; He *et al*., 2017). Both reference strains (CB1190 or PH‐06) and the two consortia were grown in AMS medium with dioxane as the only carbon source, and were harvested in exponential stage by centrifuging and washing with AMS medium for three times. Batch biodegradation assays were conducted in 250 ml bottles amended with 100 ml of AMS medium with an initial dioxane concentration of 500 mg l^−1^, while shaking similarly on a Digital Linear Shaker (Scilogex) at 150 rpm. The initial biomass concentrations (measured as protein content) were 25 mg l^−1^ for CB1190, 32 mg l^−1^ for PH‐06, 19 mg l^−1^ for Consortium A and 30 mg l^−1^ for Consortium B.

Microcosms prepared with dioxane‐impacted groundwater from a site in Seattle, WA, USA, that was not exhibiting intrinsic biodegradation capabilities. A low initial concentration of dioxane (300 μg l^−1^) was used to assess the potential of these consortia for bioaugmentation of contaminated sites where dioxane might be present at insufficient concentrations to induce and/or sustain an active degrading indigenous community. The concentrations of cocontaminants in the groundwater, such as chlorinated solvents and BTEX, were very low (< 6 μg l^−1^ for organic contaminants and < 15 mg l^−1^ for heavy metals) or non‐detectable (Table S1). The initial bacterial biomass concentrations in this study were 20 mg l^−1^ for CB1190, 35 mg l^−1^ for PH‐06, 48 mg l^−1^ for Consortium A and 18 mg l^−1^ for Consortium B. This study was conducted at 15°C to mimic the actual average groundwater temperature.

### Dioxane and biomass analytical procedures

To measure the dioxane concentrations in the original soil samples, 5 g of soil was mixed with 10 ml of deionized water and vortexed vigorously on a Vortex‐Genie^®^ 2 (MO BIO Laboratories, Inc.) at the maximum speed for 10 min. The extracted dioxane concentration in the aqueous phase was measured as detailed below, and the total dioxane mass contained in the 10 ml of extract was divided by the initial 5 g of soil to calculate the soil concentration.

Liquid samples were filtered through 0.22 μm syringe filters, and dioxane was extracted by the liquid/liquid frozen micro‐extraction method using dichloromethane as the solvent (Li *et al*., 2011). Dioxane concentrations were measured using an Agilent 7820A gas chromatograph equipped with a 5977E mass spectrum detector.

Total biomass was quantified as the total protein concentration using the Pierce™ BCA Protein Assay Kit (Thermo Fisher Scientific, Rockford, IL, USA). Serial dilutions of the bovine serum albumin (BSA) standard were prepared for calibration.

Bacterial growth on dioxane was assessed per increase in optical density at 600 nm (OD_600_). Even though both consortia clumped to a much lower extent than the two archetype dioxane degraders (CB1190 and PH‐06), the bacterial suspensions were not as uniform as that of other non‐aggregating bacteria. Thus, before the measurement of OD_600_, the bacterial cultures were put into a 1.5 ml centrifuge tube and sonicated for 10 min in a 5510 Branson Ultrasonic Cleaner (Sigma‐Aldrich) at 40 kHZ. The OD_600_ was then measured with a Ultrospec™ 2100 *pro* UV/Visible Spectrophotometer (GE Healthcare, Little Chalfont, UK).

### Microbial community analyses

16S rRNA gene sequencing was used to determine the microbial community composition of the original soil samples and the enriched two consortia. DNA from the original soil samples was extracted using PowerSoil^®^ DNA Isolation Kit (MO BIO Laboratories, Inc.). DNA from bacteria was extracted from bacteria biomass harvested in exponential growth phase, when half or more of the added dioxane (100 mg l^−1^) was consumed. The extraction kit was the UltraClean^®^ Microbial DNA Isolation Kit (MO BIO, Carlsbad, CA, USA). The V4 region of the 16S rRNA gene was amplified by PCR using the forward 515F and reverse 806R primers (Caporaso *et al*., 2011). Sequencing was performed at MR DNA (www.mrdnalab.com, Shallowater, TX, USA) by Illumina MiSeq paired‐end sequencing (approximately 2 × 300 bp as the read length). Sequence data were processed using MR DNA analysis pipeline. Operational taxonomic units (OTUs) were defined by clustering at 3% divergence (97% similarity) (Konstantinidis and Tiedje, 2005). Final OTUs were taxonomically classified using BLASTn against the RDPII (http://rdp.cme.msu.edu) and NCBI (www.ncbi.nlm.nih.gov ) databases.

### Real‐time quantitative PCR (qPCR)

qPCR analysis of the bacterial DNA was conducted to determine the presence and abundance of 16S rRNA and *thmA/dxmA* genes (Li *et al*., 2013a). 16S rRNA was used as a positive control to show amplification of genes unrelated to degradation of dioxane. qPCR mixture contained 10 ng of genomic DNA, 300 nM of forward and reverse primers, 150 nM of fluorogenic probe, 10 μl of TaqMan universal master mix II (Applied Biosystems, Foster City, CA, USA) and DNA‐free water, to a total volume of 20 μl. qPCR was performed in a CFX 96™ Real‐Time System (Bio‐Rad, Hercules, CA, USA) with the following temperature set up: 50°C for 2 min, 95°C for 10 min and 40 cycles of 95°C for 15 s and 60°C for 10 min. Serial dilutions (10^−4^ to 10 ng of DNA μl^−1^) of the extracted genomic DNA of CB1190 were used to prepare the calibration curves and determine the amplification efficiency for the tested genes.

### Analysis of soluble di‐iron monooxygenase (SDIMO) genes

Previously designed degenerate primers, NVC57, NVC58, NVC65 and NVC66, to target conserved regions in the soluble di‐iron monooxygenases (SDIMO) alpha subunit gene (Coleman *et al*., 2006) were used to examine the presence and diversity of SDIMO genes in the original soil samples and the two consortia. A nested PCR strategy was used to increase the PCR product yield. In the first run, the PCR mixture contained 1 μl of NVC65 and NVC58 primer mixture (10 μM), 20 ng of the extracted genomic DNA, 12.5 μL of KAPA HiFi HotStart ReadyMix (2X) (KAPA Biosystems, Wilmington, MA, USA) and nuclease‐free water to yield a total volume of 25 μl. PCR was performed in a Bio‐Rad T100™ Thermal Cycler (Bio‐Rad) with the following temperature profile: initial denaturation (94°C, 5 min), then 29 amplification cycles (94°C for 30 s, 55°C for 30 s, 72°C for 1 min per kb) and a final extension (72°C for 5 min) (Coleman *et al*., 2006). The length of the PCR products in the first run was checked by 1% agarose gel, and DNA bands of the correct size (1100 bp) were excised and purified. Twenty nanograms of the purified PCR product was used as the DNA template in the second run, with the second set of primers (NVC57 and NVC66). The purified product (420 bp) from the second PCR was sent to MR DNA (www.mrdnalab.com, Shallowater, TX, USA) for Illumina MiSeq paired‐end sequencing (approximately 2 × 300 bp as the read length). Sequence data were processed using MR DNA analysis pipeline. Operational taxonomic units (OTUs) were defined by clustering at 3% divergence (97% similarity) (Konstantinidis and Tiedje, 2005). A database including all of the currently reported SDIMO genes on NCBI was created and used to taxonomically classify the final OTUs.

### Statistical analysis

All treatments were conducted in triplicate. Statistical significance of differences between experimental treatments was assessed using two‐tailed unpaired Student's *t*‐test at the 95% confidence level.

## Conflict of Interest

None declared.
